# From Fin to Limb: Orientational Shift and Evolution of Diagonal-Couplet Gait in Tetrapods

**DOI:** 10.1093/iob/obag020

**Published:** 2026-05-06

**Authors:** T Miyake, K Nishikawa, M Iwata, H Kamiyama, K Ozaki, H Koie, A Yozu, T Hirasawa, N Kobayashi

**Affiliations:** Department of Medical Education, Ehime University Graduate School of Medicine, To-on, Ehime 791-0295, Japan; Graduate School of Human and Environmental Studies, Kyoto University, Yoshida Nihonmatsu-cho, Sakyo 606-8501, Kyoto, Japan; Graduate School of Global Environmental Studies, Kyoto University, Yoshida Hon-machi, Sakyo, Kyoto 606-8501, Japan; Institute for Environment and Development (LESTARI), Universiti Kebangsaan, Malaysia, UKM Bangi, Selangor 43600, Malaysia; Department of Biology, Faculty of Science, Chulalongkorn University, Bangkok 10330, Thailand; Aquamarine Inawashiro, Kingfishers Aquarium, Osada, Inawashiro-machi, Fukushima 969-3283, Japan; Atagawa Tropical & Alligator Garden, Naramoto Higashiizu-cho, Shizuoka 413-0302, Japan; Graduate School of Human and Environmental Studies, Kyoto University, Yoshida Nihonmatsu-cho, Sakyo 606-8501, Kyoto, Japan; Laboratory of Veterinary Physiology, Department of Veterinary Medicine, College of Bioresource Sciences, Nihon University, Fujisawa 252-0880, Japan; Department of Rehabilitation Medicine, Nippon Medical School, Sendagi, Tokyo 113-8602, Japan; Department of Earth and Planetary Environmental Science, School of Science, The University of Tokyo, Tokyo 113-0033, Japan; Department of Medical Education, Ehime University Graduate School of Medicine, To-on, Ehime 791-0295, Japan

## Abstract

The transition from fins to limbs represents a pivotal evolutionary shift that enabled vertebrates to engage terrestrial environments through coordinated transformations in appendicular structure and neuromuscular control. Here, we propose that this transition can be understood as a biomechanical reorganization of force transmission across multi-joint musculoskeletal linkages. Rather than a simple rotational modification transforming fins directly into forelimbs, this process involved progressive reorganization of sarcopterygian appendages, including axial rotation, directional force production, and coordinated stance-swing dynamics that likely emerged prior to fully terrestrial locomotion. This review applies the two-joint link model, originally developed for human limb biomechanics, to extant sarcopterygians such as the coelacanth (*Latimeria*) and to early-diverging tetrapods, including archosaurs such as the American alligator (*Alligator mississippiensis*). The model demonstrates how sequential activation of biarticular and monoarticular muscles generates predictable, axis-aligned endpoint force outputs, providing a mechanistic explanation for how ancestral fins and early limbs could support weight bearing, propulsion, and postural stabilization during the water-to-land transition. The framework further clarifies the evolution and persistence of diagonal-couplet lateral sequence gait, a locomotor pattern widespread among tetrapods and consistent with Paleozoic trackway evidence. Integration of electromyographic data with conserved spinal circuitry including central pattern generators, interneurons, and the topographic organization of lateral motor column motor pools reveals how intrinsic spinal architecture governs muscle activation sequences and limb-level force redirection. Although supraspinal pathways modulate locomotion, core coordination of diagonal-couplet gait emerges from spinal mechanisms. A central property of the model is musculoskeletal redundancy: multiple muscle combinations can generate equivalent endpoint forces within linked multi-joint systems, thereby preserving functional capacity across postural variation. This robustness arises from the four-bar linkage organization of biarticular muscles, a design principle conserved across vertebrate musculoskeletal systems. Together, these findings position the two-joint link model as a unifying neuromechanical framework for understanding fin-to-limb evolution, the emergence of tetrapod gait, and the conserved spinal organization underlying vertebrate locomotion.

## Japanese

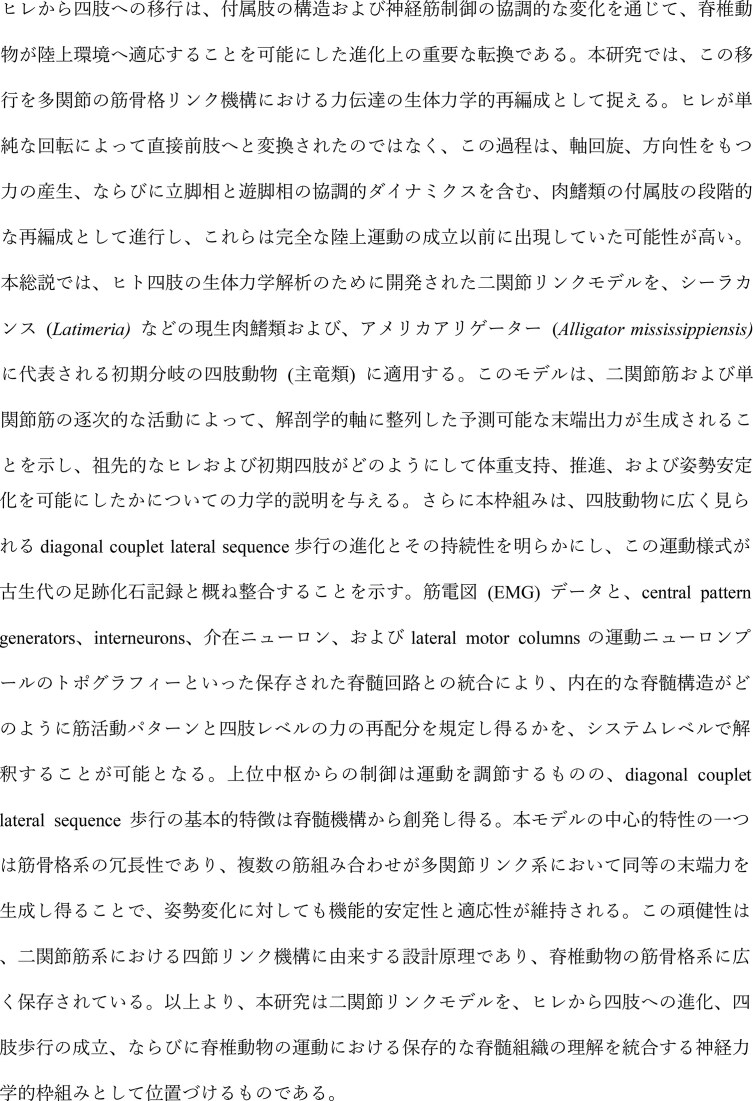

## Introduction

The transition from fins to limbs represents one of the most transformative events in vertebrate evolution, enabling sustained interaction with terrestrial substrates through coordinated changes in appendicular orientation, force transmission, and neuromuscular control (Hall 2006; Clack 2009). Rather than a sudden innovation, this shift reflects a gradual reorganization of paired appendages in sarcopterygian fishes, involving axial rotation, directional force production, and coordinated stance-swing dynamics that likely emerged prior to fully terrestrial locomotion (Boisvert 2005; Shubin et al. 2006; Molnar et al. 2026). Recent developmental evidence further indicates that dorsoventral limb patterning arose through regulatory repurposing of an ancestral posterior fin module, linking morphological transformation to shifts in gene regulatory networks (Zdra et al. 2025). Accordingly, understanding how ancestral fins generated, oriented, and redistributed force is central to explaining the mechanical origins of tetrapod weight-bearing and propulsion.

To address this problem, we apply the two-joint link model of biarticular and monoarticular muscle coordination as a unifying biomechanical framework. Originally developed for human limb analysis (Fujikawa et al. 1996; Oshima 2008), the model has since been extended to comparative, cross-species contexts (Miyake and Okabe 2022; Miyake et al. 2024; Miyake and Yozu 2025; Miyake et al. 2026). Within this framework, sequential muscle activation is organized into predictable activity switches that generate combined force outputs aligned with anatomical axes, while musculoskeletal redundancy stabilizes force output across changing joint configurations. In addition, proximal orientational changes are systematically translated into distal, task-level force regulation, providing a functional bridge between morphology and mechanics. By emphasizing sectoral force progression rather than isolated joint torques, the framework provides a systems-level interpretation of appendicular function (Miyake and Okabe 2022; Miyake et al. 2024).

Applied to extant sarcopterygians such as *Latimeria* and to early-diverging tetrapods and archosaurs including the American alligator (*Alligator mississippiensis*), the model clarifies how ancestral fin and limb architectures supported axial rotation, effective force redirection, and contralateral limb coupling (Fricke and Hissmann 1992; Gatesy 1997; Iwata 2017). These features foreshadow the mechanical organization of tetrapod limbs, indicating functional continuity between aquatic fin-based locomotion and terrestrial gait.

**Fig. 1:**
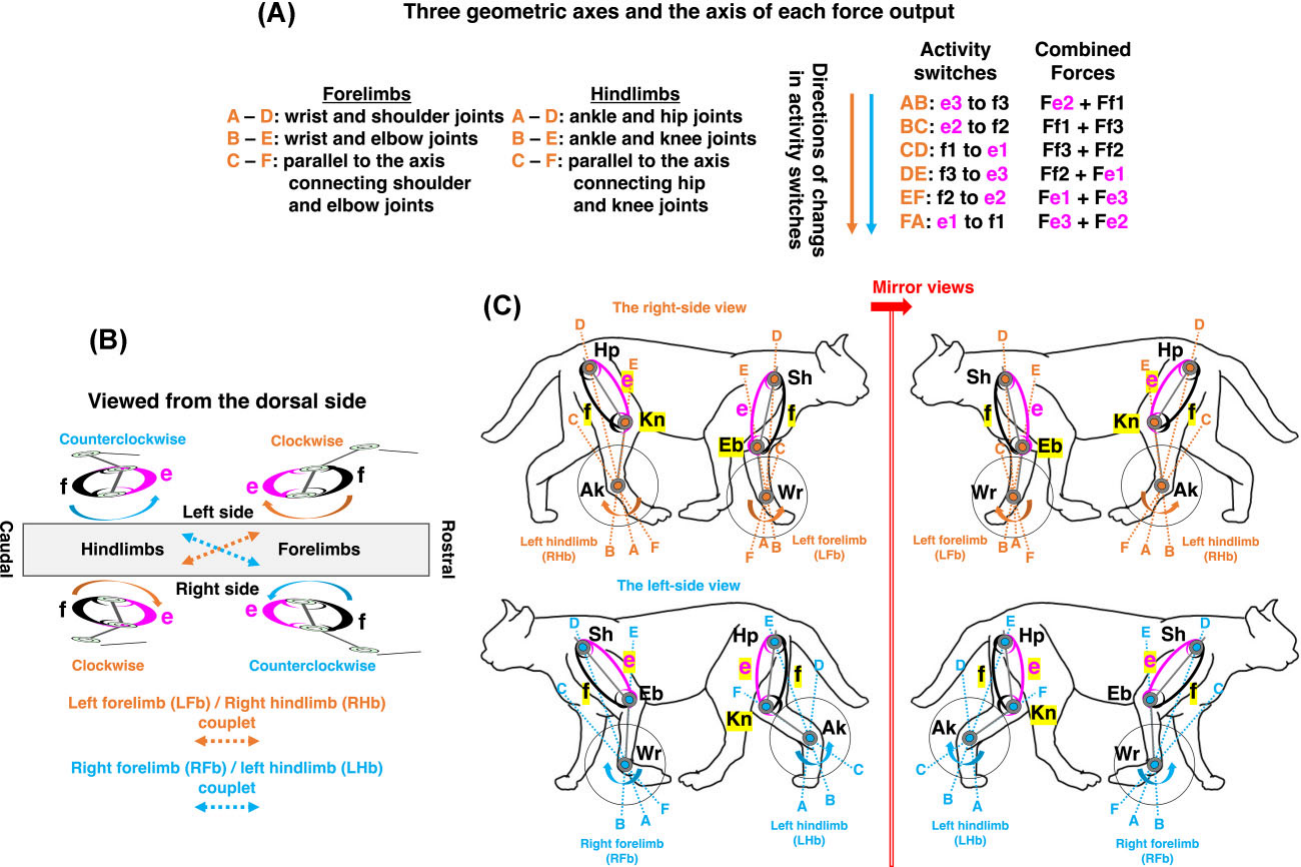
The two-joint link model of biarticular and monoarticular muscles in tetrapod limbs. (A) Functional organization of muscles in the two-joint link model, illustrating three orthogonal axes in forelimbs and hindlimbs, activity switches, and corresponding combined force outputs. All biological characteristics of the model follow Miyake and Okabe (2022), Miyake et al. (2024), Miyake and Yozu (2025), and Miyake et al. (2026). (B) Directional changes in combined force outputs in a quadrupedal model. In the left forelimb-right hindlimb couplet (LFb-RHb), the change occurs clockwise, whereas in the right forelimb-left hindlimb couplet (RFb-LHb), the change occurs counterclockwise. (C) A schematic presentation of directional changes in combined force outputs associated with activity switches, viewed in the sagittal plane (right and left views shown as mirror images). Rotation is clockwise in the left forelimb-right hindlimb couplet (LFb-RHb) and counterclockwise in the right forelimb-left hindlimb couplet (RFb-LHb). All transitions proceed from the f-series toward the e-series muscle groups. Ak: ankle joint; Eb: elbow joint; Hp: hip joint; Sh: shoulder joint; Wr: wrist joint.

Importantly, the framework integrates biomechanics with neuromuscular organization. Within this framework, coordinated muscle activity can be understood as a dorsoventral “switching” pattern, in which dorsal (e-series) and ventral (f-series) muscle groups alternate their functional roles across forelimbs and hindlimbs to generate coordinated, coupled limb movements (Fig. 1). Electromyographic (EMG) studies in mammals demonstrate conserved dorsoventral muscle compartmentalization and diagonal-couplet coordination consistent with predicted e-series and f-series activity switches (Miyake et al. 2024; Miyake and Yozu 2025; Miyake et al. 2026), while reptilian studies document coordinated interlimb coupling and stance-swing organization (Gatesy 1997; Reilly and Elias 1998; Reilly et al. 2005; Iijima et al. 2024). These patterns are consistent with the organization of spinal motor circuits, including lateral motor column architecture and central pattern generator networks, which together structure rhythmic and task-specific muscle activation. These findings indicate that locomotor output arises from structured muscle switching embedded within conserved spinal circuits rather than from isolated joint actions (Fetcho 1987; Rossignol et al. 2006; Murakami and Tanaka 2011; Grillner and Manira 2020). This perspective provides a mechanistic basis for the persistence of diagonal-couplet lateral sequence gait across vertebrate evolution, as supported by extant taxa and Paleozoic trackways (Niedźwiedzki et al. 2010; Nyakatura et al. 2019; Struble and Gibb 2022).

Grounded in comparative anatomy, functional biomechanics, developmental genetics, and electromyographic (EMG)-based inference, this review proposes that the fin-to-limb transition reflects an integrated reorganization of appendicular polarity, rotational capacity, and force-output control. By explicitly linking musculoskeletal mechanics with underlying spinal circuit organization, we argue that biomechanical architecture and neuromuscular coordination evolved in concert to shape the emergence and stabilization of tetrapod locomotion.

## The two-joint link model of biarticular and monoarticular muscles

The two-joint link model provides a biomechanical framework for understanding the coordinated muscle-force redirection in tetrapod limbs. Originally developed for human limbs, the model analyzes the functional roles of biarticular and monoarticular muscles through experimental and theoretical investigations (Fujikawa et al. 1996; Fujikawa et al. 1997; Oshima et al. 1999). Initially based on static models, it predicts dynamic force outputs at the distal joint independent of conventional moment-arm analysis, utilizing isometric push-pull tasks and integrated electromyographic (IEMG) data (Fujikawa et al. 1996; Fujikawa et al. 1997; Fujikawa et al. 2001). To extend this framework to tetrapods, Miyake and Okabe (2022) generalized the model’s anatomical configuration for both forelimbs and hindlimbs. The model defines three antagonistic muscle pairs: (1) monoarticular muscles acting at the proximal hip joint (e1/f1), (2) monoarticular muscles acting at the distal knee joint (e2/f2), and (3) biarticular muscles spanning both the hip and knee joints (e3/f3) (Fig. 1A). These muscle groups interact along three orthogonal axes (A-D, B-E, C-F) at the wrist or ankle, creating six sectors that represent force direction domains (Miyake et al. 2024; Miyake and Yozu 2025; Miyake et al. 2026) (Fig. 1A).

Sequential switching between these muscle pairs rotates the combined force outputs across adjacent sectors, generating a cyclic redirection of force (Fig. 1A). The model predicts that this rotational change of combined force outputs occurs clockwise in the left forelimb (LFb) and the right hindlimb (RHb) but counterclockwise in the right forelimb (RFb) and the left hindlimb (LHb) (Miyake et al. 2024; Miyake and Yozu 2025; Miyake et al. 2026) (Fig. 1B). Miyake et al. (2026) demonstrated that, in the sagittal plane (right-side view), resultant distal joint force outputs exhibit a diagonally coupled coordination pattern: clockwise rotation in the left forelimb (LFb) and right hindlimb (RHb), and counterclockwise rotation in the right forelimb (RFb) and left hindlimb (LHb) (Fig. 1C). This pattern is consistent with the flexing direction of the right elbow joint and the right knee joint, preserving a conserved limb-axis orientation that aligns with the functional coupling of diagonally opposite limbs (Miyake et al. 2024; Miyake and Yozu 2025; Miyake et al. 2026). This diagonally coordinated force organization provides a biomechanical basis for interpreting how limb movement remain functionally integrated across contralateral forelimb-hindlimb pairs. Such conserved coordination pattern offers a useful framework for examining how appendicular force organization may have been maintained during the evolutionary transition from paired fins to weight-bearing limbs.

Although initially derived from static experiments, the model predicts dynamic force patterns consistent with electromyographic (EMG) data from human pedaling, walking, sprinting, and quadrupedal walking in mammals such as cats, dogs, and horses (Fujikawa et al. 2010; Miyake et al. 2024; Miyake and Yozu 2025; Miyake et al. 2026). These patterns reflect the rotational progression of force between muscle groups, which is reversed between diagonal limb pairs (Fig. 1B, C). As muscle activity switches during locomotion, the force orientation progresses systematically, ensuring coordination between the limbs (Miyake and Yozu 2025; Miyake et al. 2026). Collectively, the two-joint link model offers a mechanistic understanding of how coordinated muscle forces are redirected during tetrapod locomotion. It serves as the biomechanical basis for interpreting how conserved muscle coordination patterns were likely reorganized during the fin-to-limb transition, particularly in relation to the dorsoventral repatterning and the emergence of diagonal-couplet locomotion.

## The dorsoventral organization and rotational reorientation during fin-to-limb transition

### Metapterygial architecture and asymmetric forelimb rotation

**Fig. 2:**
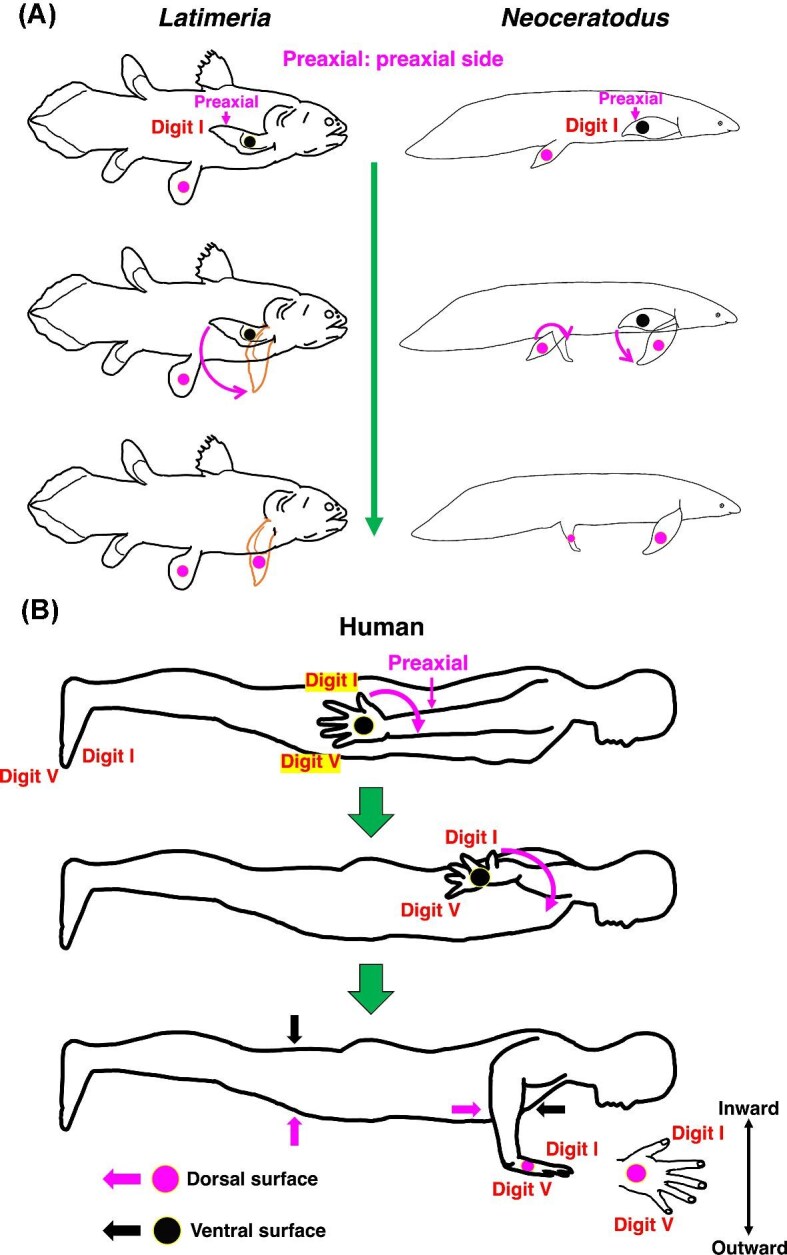
Proposed orientational transformation of fish paired fins to tetrapod limbs. (A) In the coelacanth *Latimeria* and lungfish *Neoceratodus*, the pelvic fins are oriented with their dorsal surfaces facing upward without requiring rotation. In contrast, the pectoral fin must rotate so that its dorsal surface faces upward. (B) Mimicking of orientational transformation. If a human mimics this rotational, orientational transformation of the right pectoral fin, the resulting posture resembles lying on the ground with the hand positioned beneath the body, the first digit pointing inward and the fifth digit outward. The preaxial side of the pecotral fin and forelimb is indicated.

The forelimb of sarcopterygians and tetrapods comprises the stylopodium (humerus) and zeugopodium (radius on the side and ulna on the postaxial side) organized along a single metapterygial axis (Shubin and Alberch 1986; Trofka et al. 2021; Jia et al. 2022). In the African coelacanth (*Latimeria chalumnae*), the pectoral fin includes homologous elements, with the preaxial side positioned dorsally and the postaxial side ventrally in the resting orientation (Miyake et al. 2016) (Fig. 2A). The establishment of distinct dorsal and ventral appendicular surfaces in early vertebrates has been proposed as a key prerequisite for fin-to-limb transformation (Miyake and Okabe 2022).

The transition from fins to limbs involved an asymmetric rotational requirement between appendages. Pelvic fins were already oriented with the dorsal surface facing upward and thus required minimal reorientation during hindlimb evolution, whereas pectoral fins required substantial long-axis rotation to reposition the dorsal surface upward and align the preaxial side rostrally (Romer 1971; Hall 2006; Clack 2009; Miyake and Okabe 2022) (Fig. 2A). This asymmetry indicates that the primary mechanical challenge of appendicular reorientation was concentrated in the forelimb lineage. In humans, mimicking this rotation of the right forelimb results in the hand being oriented with the first digit directed medially and the fifth digit laterally beneath the body, providing a simple analogue of the rotational transformation underlying the pectoral fin-to-forelimb transition (Fig. 2B).

**Fig. 3:**
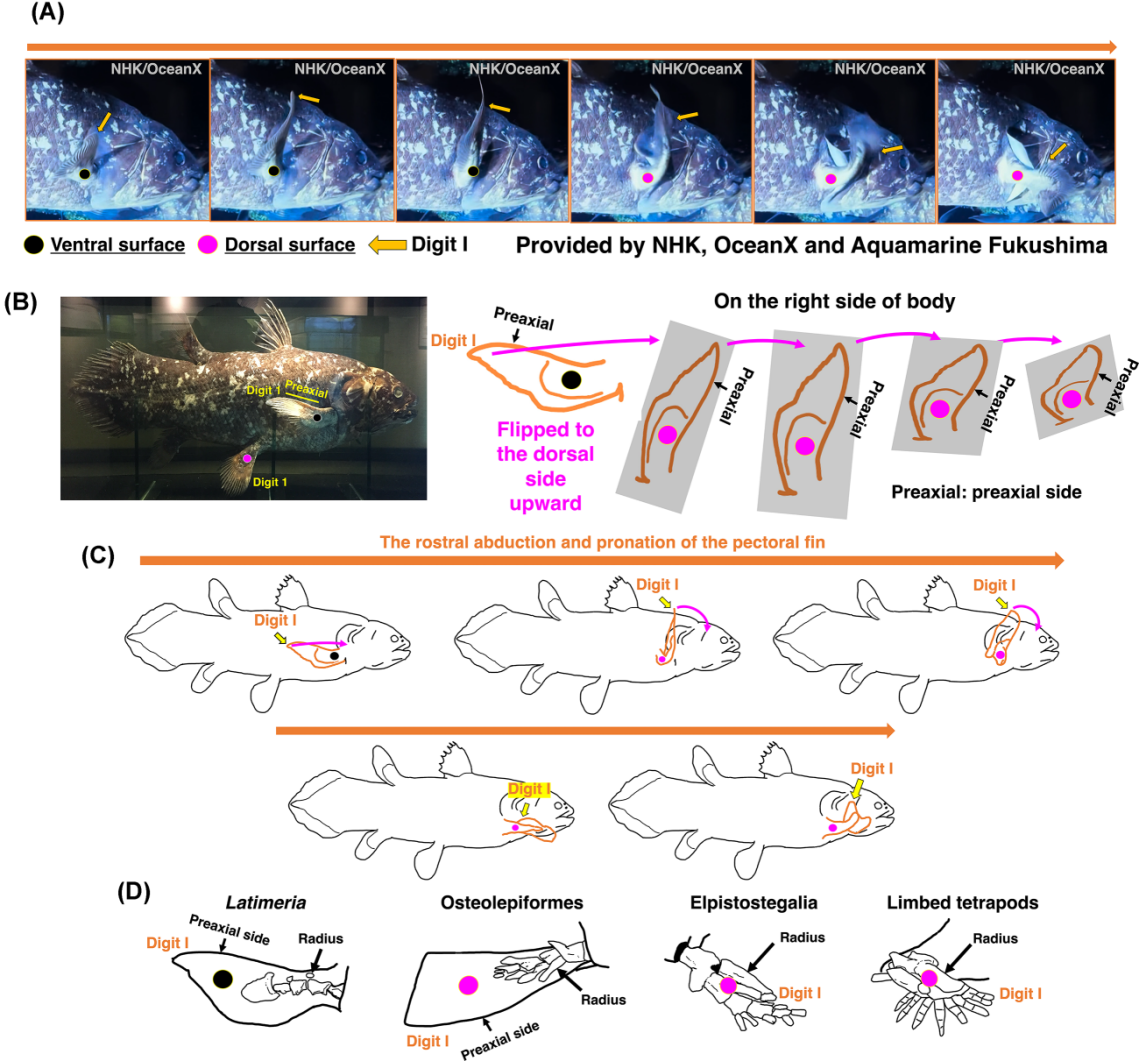
The actual rotation of the pectoral fin in the coelacanth *Latimeria menadoensis*. (A) Six video frames showing the rostral rotation of the pectora fin *in situ*, illustrating the orientational transformation proposed in the present study. The footage was provided by the Japan Broadcasting Corporation (NHK), OceanX and Aquamarine Fukushima, Japan. The preaxial side of the pectoral fin is indicated. (B) Traced rotational movement of the pectoral fin based on the video analysis. Note the position of the preaxial side where the first “digit” is located. (C) Schematic representation of the fin’s rotational movement *in situ*, reconstructed from the video frames. Note the position of the preaxial side where the first “digit” is located. Once the fin was completely flipped, it was immediately flipped back to the resting position. (D) Proposed rotation of pectoral fins from sarcopterygians (e.g., *Latimeria*), through osteolepiforms (e.g., *Eusthenopteron*) and elpistostegalians (e.g., *Tiktaalik*), to forelimbs of early limbed tetrapods (e.g., *Acanthostega*) during the fin-to-limb transition (Boisvert 2005; Daeschler et al. 2006; Clack 2009; Pierce et al. 2012; Pierce et al. 2013). In the transition from sarcopterygians to osteolepiforms, pectoral fins were required to face dorsally, positioning the preaxial side rostrally and thus the first digit rostrally and inward.

In sarcopterygian fishes such as *Latimeria chalumnae* and the lungfish *Neoceratodus forsteri*, the metapterygial axis permits considerable rotational mobility (Boisvert et al. 2008; Miyake et al. 2016; Miyake and Okabe 2022), and shoulder joint morphology in *Latimeria* does not osteologically constrain long-axis rotation (Miyake et al. 2016; Iwata 2017). Observations of *Latimeria menadoensis* based on *in situ* deep-sea video recordings and analyses of an actual specimen further demonstrate dynamic pectoral fin movements involving abduction and pronation (Fig. 3A, B, C), producing a transition from the resting orientation to a rostro-dorsally elevated posture resembling humeral long-axis rotation in early tetrapods (Molnar et al. 2021; Demuth et al. 2023). These movements reposition the preaxial side rostrally, thereby supporting the hypothesis that rotational reorientation facilitates forelimb positioning for substrate interaction (Miyake and Okabe 2022) (Fig. 3A, B, C).

In contrast, early terrestrial tetrapods likely exhibited more restricted humeral long-axis rotation based on joint morphology and functional reconstructions (Clack 2009; Pierce et al. 2013: Nyakatura et al. 2019; Molnar et al. 2026) (Fig. 3D), indicating that rotational mobility may have been constrained in association with the demands of stable weight-bearing function. However, rotation alone cannot fully account for the establishment of functional limb polarity. Developmental evidence indicates that dorsoventral limb patterning arose through regulatory redeployment of an ancestral posterior fin module (Zdra et al. 2025), reinforcing intrinsic appendicular polarity (Hall 2006). Following axial rotation, this reorganization establishes correspondence between the preaxial side and the ventral domain, and between the postaxial side and the dorsal domain, as observed in *Latimeria menadoensis* (Fig. 3A, B, C). Notably, the resulting configuration aligns with predictions of the two-joint link model, in which f-series muscles are functionally associated with the preaxial domain and e-series muscles with the postaxial domain in the forelimb (Fig. 1C). Together, appendicular reorientation and dorsoventral patterning establish the functional muscle organization required for coordinated limb-level force generation.

### Fossil evidence for preaxial reorientation

Although fossil evidence provides limited direct information on gait patterns and locomotor mechanics, it nonetheless offers important insights into appendicular orientation and structural reorganization during the fin-to-limb transition. Fossil evidence from Devonian tetrapodomorphs offers critical insights into the early stages of preaxial reorientation. In taxa such as *Eusthenopteron foordi, Panderichthys rhombolepis, Tiktaalik roseae*, and *Elpistostege watsoni*, the pectoral endoskeleton exhibits a rostrally oriented preaxial margin, positioning digit-like elements anteriorly and indicating forward rotation of the fin relative to the trunk (Daeschler et al. 2006; Boisvert et al. 2008; Pierce et al. 2013) (Fig. 3D). This forward rotation suggests progressive reorientation of the appendage, likely contributing to the establishment of functional forelimbs in early tetrapods (Clack 2009; Miyake and Okabe 2022). Among early limbed tetrapods such as *Acanthostega gunnari, Ichthyostega stensioei*, and *Tulerpeton curtum*, this rostral preaxial orientation was maintained, further supporting the view that pectoral fin rotation played a pivotal role in the evolution of functional forelimbs (Lebedev and Coates 1995; Clack 2009). These data also suggest that shoulder protraction, retraction, and elbow flexion-extension became integral to substrate-based locomotion in these early tetrapods (Pierce et al. 2012; Pierce et al. 2013; Nyakatura et al. 2019).

### Diagonal-couplet coordination in the coelacanth *Latimeria*

**Fig. 4:**
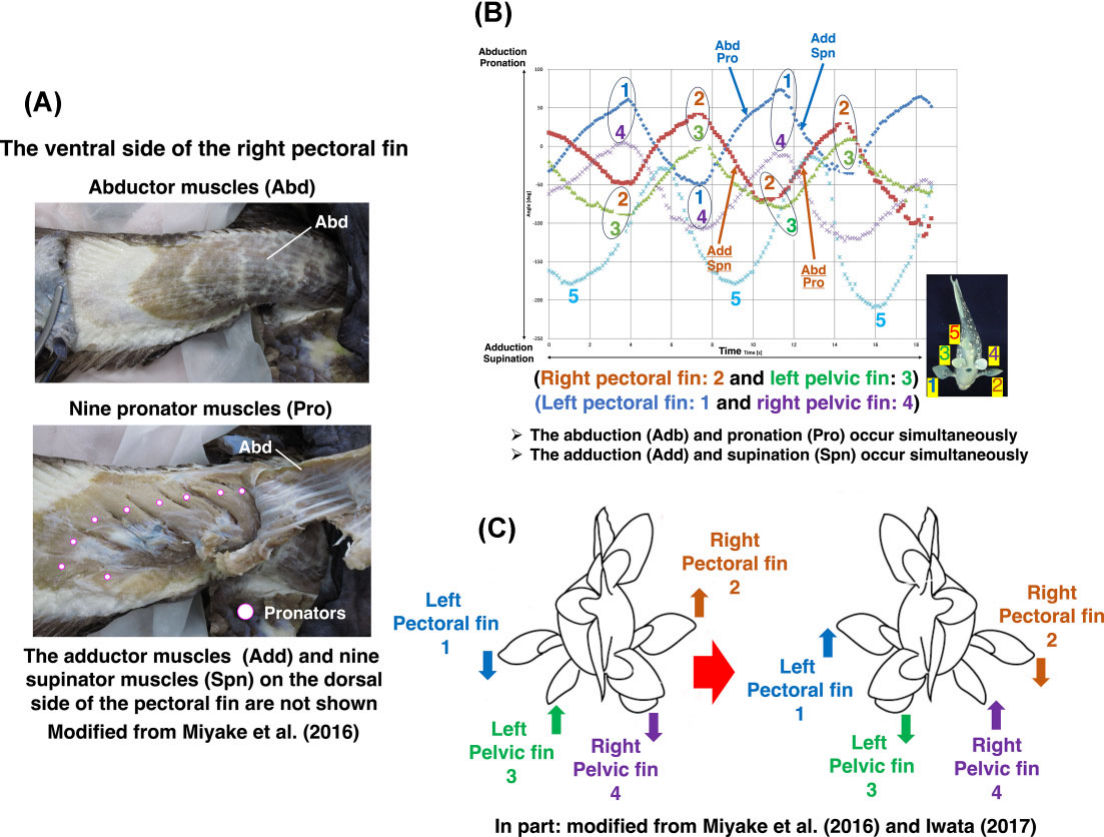
Couplet movements of the pectoral and pelvic fins in the coelacanth *Latimeria chalumnae* and *Latimeria menadoensis*. Modified from Miyake et al. (2016) and Iwata (2017). (A) Musculature of the right pectoral fin in *L. chalumnae.* The superficial abductor muscle (Abd) on the ventral domain consists of two layers: superficial and profundus. Beneath the Abd are nine pronator muscles (Pro) that span two or more joints along the metapterygium. On the dorsal domain, one adductor muscle (Add) and nine supinator muscles (Spn) are present (not shown). Together with the antagonistic adductor and supinator muscles on the dorsal domain, the ventral muscles generate a figure-eight trajectory at the distal tip of the fin (Miyake et al. 2016; Iwata 2017). (B) Visualization of the coupled movements of the right pectoral fin (2) with the left pelvic fin (3) and the left pectoral fin (1) with the right pelvic fin (4). The movement of each pectoral fin is produced by synchronized activity of a pair of adductor (Add) and supinator (Spn) muscles and a pair of abductor (Abd) and pronator (Pro) muscles. The synchronized activation of these antagonistic pairs in the right and left pectoral fins generates opposite directional movements of the fins. The alternating movements of the two pectoral and pelvic fin couplets resemble the diagonal-couplet lateral sequence gait of tetrapods. The x-axis is the time for fin movements; the y-axis is a degree of abduction/pronation of the pectoral fin or adduction/supination of the pectoral fin. (C) Two synchronized pectoral and pelvic fin couplets during swimming in *Latimeria. menadoensis* (Iwata 2017): the left pectoral fin with the right pelvic fin (1 and 4) and the right pectoral fin with the left pelvic fin (2 and 3). These coordinated fin movements represent a sarcopterygian analogue of the diagonal-couplet lateral sequence gait observed in tetrapods. Supplementary data 1.

Evidence for diagonal coordination predates terrestrial tetrapods and is clearly documented in extant sarcopterygians. Miyake et al. (2016) described the detailed organization of biarticular and monoarticular muscles in the pectoral fins of the African coelacanth (*Latimeria chalumnae*). Each pectoral fin comprises a large abductor muscle (Abd) spanning the entire fin, beneath which nine pronator muscles (Pro) are arranged on the ventral domain. On the dorsal domain, an antagonistic adductor muscle (Add) similarly spans the fin, accompanied by nine supinator muscles (Spn) located beneath the adductor (Miyake et al. 2016) (Fig. 4A). This clear dorsoventral antagonistic organization establishes a functional architecture comparable to limb-level abductor-adductor and rotator systems.

Building upon this anatomical framework, Iwata (2017) analyzed swimming biomechanics in *Latimeria chalumnae* and *L. menadoensis* using *in situ* deep-sea video recordings (Fig. 4B, C). These observations revealed coordinated fin movements organized in a diagonal-couplet pattern. Coordinated movements of the contralateral pectoral fins (1 and 2), pelvic fins (3 and 4), and the anal fin (5) revealed a distinct diagonal-couplet pattern. When the left pectoral fin (1) reaches its lowest position, the right pectoral fin (2) is positioned highest. Simultaneously, the left pelvic fin (3) is elevated, whereas the right pelvic fin (4) is depressed (Fig. 4C). In the subsequent phase, the left pectoral fin (1) and right pelvic fin (4) elevate together, while the right pectoral fin (2) and left pelvic fin (3) move downward (Iwata 2017) (Fig. 4C). Thus, the right pectoral fin (2) and left pelvic fin (3) form one functional couplet, whereas the left pectoral fin (1) and right pelvic fin (4) form the alternating couplet (Fig. 4B).

The coordinated pattern of alternating diagonal fin couplets closely parallels the diagonal-couplet lateral sequence gait observed in tetrapods. Synchronized pectoral-pelvic fin activity has been documented during slow forward swimming, drifting, hovering, upside-down swimming, and head-standing behaviors (Fricke and Hissmann 1992; Iwata 2017). Comparable diagonal coordination has also been reported in the waterfall-climbing cave fish *Cryptotora thamicola*, where teleost locomotion exhibits a limb-like coupling pattern (Flammang et al. 2016; Struble and Gibb 2022). The occurrence of diagonal fin couplets in the extant sarcopterygian *Latimeria*, together with similar coordination in certain teleosts, suggests that diagonal-couplet lateral sequence organization is not a strictly terrestrial innovation (Flammang et al. 2016; Struble and Gibb 2022). Rather, it likely represents a deeply rooted vertebrate locomotor strategy that predates fully developed limbs and was retained and elaborated during tetrapod evolution (Miyake et al. 2016; Struble and Gibb 2022).

### Trackway evidence for early tetrapod locomotion

Fossil trackways from the Devonian (∼395 Ma) and Middle Permian provide early evidence of diagonal-couplet-like walking patterns in stem tetrapods (Niedźwiedzki et al. 2010; Nyakatura et al. 2019). In the Devonian record, closely spaced ipsilateral fore- and hindfoot impressions are consistent with alternating diagonal support phases. Similarly, the reptile-like amphibian *Orobates pabsti* from the Middle Permian has been reconstructed as employing alternating forelimb-hindlimb coordination consistent with diagonal-couplet-like lateral sequence gait (Nyakatura et al. 2019). Notably, the reverse-engineering approach applied to *Orobates* addressed not only limb phasing but also trunk posture and joint orientation, highlighting the broader evolutionary transition from sprawling to more upright, parasagittal configurations.

One limitation of trackway-based reconstruction is that it provides indirect evidence of limb coordination and cannot directly resolve underlying locomotor mechanics. However, such evidence remains informative when interpreted alongside extant analogs. To address this, locomotor patterns in extant tetrapods have been used as functional analogs. Crocodilians, including the American alligator (*Alligator mississippiensis*), are particularly informative due to their relatively conserved body plan, limb posture, and predominant use of diagonal-couplet locomotion during steady walking (Wimberly et al. 2021).

**Fig. 5:**
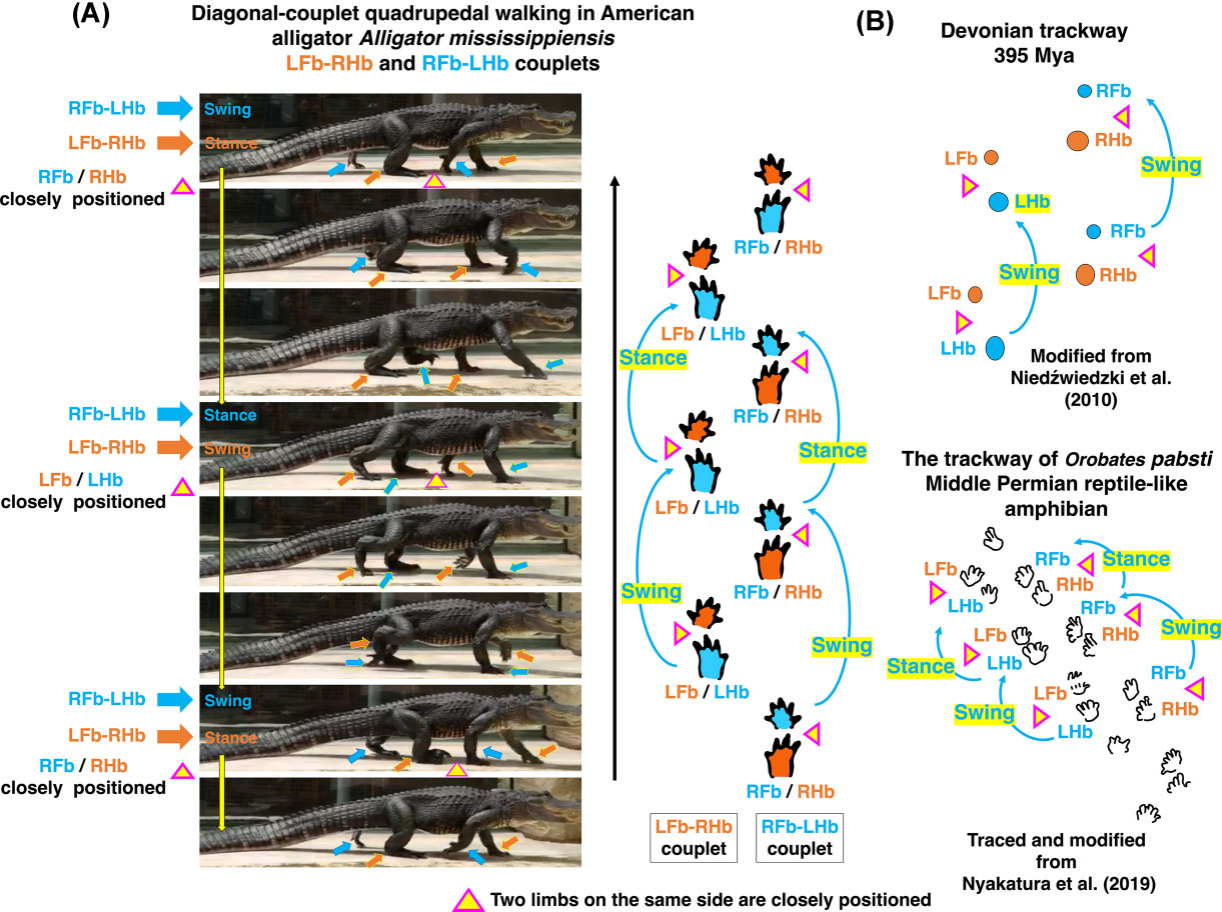
Diagonal-couplet lateral sequence gait in American alligators and fossil trackways. Video footage of an American alligator (*Alligator mississippiensis*) was recorded at Atagawa Tropical & Alligator Garden, Shizuoka, Japan. (A) Quadrupedal walking in *A. mississippiensis*, illustrating the diagonal-couplet movements: the left forelimb (LFb) coordinated with the right hindlimb (RHb), and the right forelimb (RFb) coordinated with the left hindlimb (LHb). (B) Two fossil trackways: a Devonian trackway (395 Mya) and the trackway of the Middle Permian reptile-like amphibian *Orobates pabsti*. The limb movements inferred from these trackways can be compared to the diagonal-couplet gait observed in the alligator. Supplementary data 2.

Comparative observations of quadrupedal walking in the American alligator (*Alligator mississippiensis*) reveal alternating diagonal couplets between the right forelimb-left hindlimb (RFb-LHb) and left forelimb-right hindlimb (LFb-RHb) during swing and stance phases (Wimberly et al. 2021). Each diagonal couplet undergoes swing and stance phases synchronously, such that when one couplet (LFb-RHb) is in stance, the opposite couplet (RFb-LHb) is in swing, and vice versa (Fig. 5A). As a result, ipsilateral forelimb and hindlimb (LFb and LHb or RFb and RHb) become positioned closely together during phase transitions (Fig. 5A, triangle marks). The positioning of the ipsilateral forelimb and hindlimb closely together can be explained by the action of e-series muscles on the dorsal domain of the limbs (Miyake and Okabe 2022; Miyake et al. 2026) (Fig. 1C). Similar coordination has been inferred from fossil evidence and may inform locomotor interpretations in early tetrapods and related taxa.

The sequential limb coordination observed in extant crocodilians provides a biomechanical template for interpreting fossil trackways of early tetrapods. In both Devonian and Middle Permian examples, closely spaced pairs of footprints are consistent with ipsilateral forelimb and hindlimb placement (e.g., LFb and LHb or RFb and RHb) (Niedźwiedzki et al. 2010; Nyakatura et al. 2019). In Devonian trackways, one diagonal couplet appears to have been in swing phase while the opposite couplet bore weight, and a comparable alternation has been reconstructed for the Middle Permian reptile-like amphibian *Orobates pabsti* (Nyakatura et al. 2019) (Fig. 5B). This pattern is consistent with diagonal-couplet-like lateral sequence coordination observed in modern crocodilians, including the American alligator, in which contralateral forelimb and hindlimb form functional couplets during locomotion (Wimberly et al. 2021) (Fig. 5B). The recurrence of this coordinated pattern across extant crocodilians and Paleozoic trackways supports the interpretation that diagonal-couplet-like coordination may represent a conserved locomotor strategy with deep evolutionary origins.

Fossil trackways further illuminate locomotor patterns in dinosaurs, particularly quadrupedal sauropods. Crocodilians (Pseudosuchia) and dinosaurs (Avemetatarsalia) share a common archosaurian ancestry dating to the Late Permian-Early Triassic (Xu et al. 2014; Baron et al. 2017; Langer et al. 2018; Ballell et al. 2022; Gônet et al. 2023). However, their limb postures diverged substantially: crocodilians retained a semi-sprawling stance, whereas dinosaurs evolved a more erect, parasagittal posture (Demuth et al. 2023). This postural transformation represents a major evolutionary shift in limb orientation, joint loading, and trunk stabilization. While the two-joint link model focuses on muscle coordination and force redirection at the limb level, it does not fully account for the biomechanical consequences of these large-scale postural transitions.

Nevertheless, applying a couplet-based framework to dinosaur trackways offers a testable approach. Limb-phase estimation and footprint spacing analysis (Lallensack and Falkingham 2022; Demuth et al. 2023) can be used to evaluate whether observed phasing patterns are consistent with diagonal-couplet-like lateral sequence walking despite differences in posture. If supported, such findings would suggest that diagonal-couplet-like coordination may represent a conserved ancestral strategy retained across major shifts in limb posture. Alternatively, differences in footprint geometry or phase relationships may indicate independent evolutionary solutions within erect dinosaurian lineages. Thus, while the two-joint link model does not directly address the full transition from sprawling to parasagittal and erect gaits, it provides a mechanistic framework for evaluating limb-phase coordination across these postural regimes. By integrating fossil trackways with extant tetrapod locomotion, the model contributes to a focused and testable interpretation of diagonal-couplet-like coordination within the broader evolutionary context of limb posture diversification.

### Model-based interpretation of muscle coordination in alligator locomotion

**Fig. 6:**
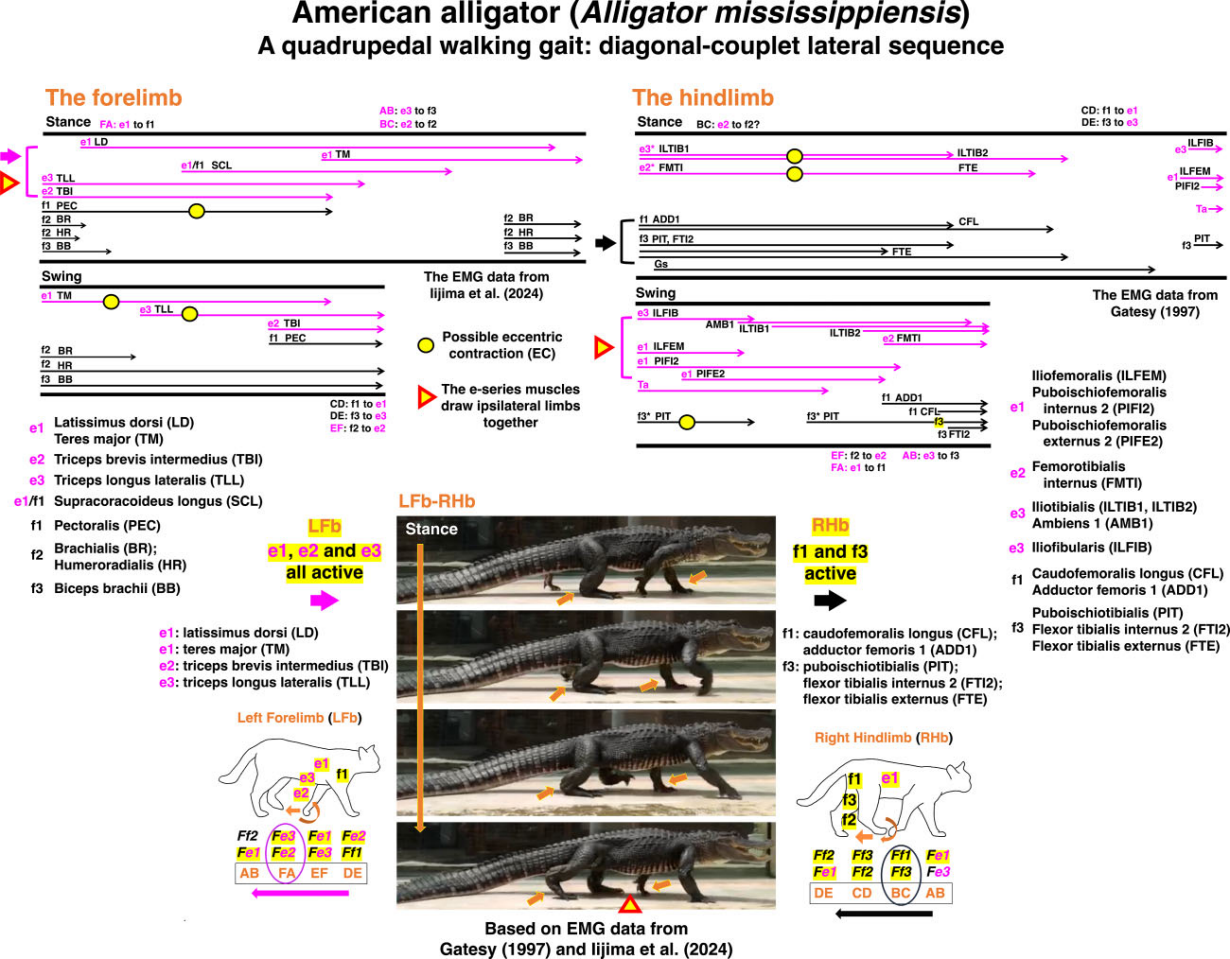
Analysis of electromyography (EMG) in forelimbs and hindlimbs during diagonal-couplet lateral sequence gait in the American alligator. Video footage of an American alligator (*Alligator mississippiensis*) was recorded at Atagawa Tropical & Alligator Garden, Shizuoka, Japan. Original EMG data on hindlimbs and forelimbs were provided by Gatesy (1997) and Iijima et al. (2024), respectively. These datasets were reanalyzed to visualize muscle activity patterns during quadrupedal walking. The muscles analyzed are listed within the figure. The e-series muscles extend the left forelimb whereas the f-series muscles extend the right hindlimb during the stance phase, demonstrating the asymmetric yet complementary contributions of the two muscle groups to couplet coordination. Proposed eccentric contractions of the muscles are indicated with circles in EMG data. Supplementary data 3.

To evaluate the functional predictions of the two-joint link model in a biological context, we examined muscle coordination in the American alligator (*Alligator mississippiensis*) using electromyographic (EMG) data (Gatesy 1997; Iijima et al. 2024). By categorizing forelimb and hindlimb muscles into e-series and f-series groups, we compared muscle activities from both EMG datasets (Fig. 6). The e-series and f-series correspond broadly to dorsal and ventral muscle domains, respectively; however, these groupings represent functional coordination within the two-joint link model rather than fixed anatomical or kinematic roles (Miyake and Okabe 2022; Miyake et al. 2024; Miyake and Yozu 2025; Miyake et al. 2026). In this framework, muscles are organized according to their contribution to coordinated force transmission across linked joints, and their apparent actions (e.g., extension or flexion) vary with limb configuration and task-specific force requirements during the locomotor cycle (Miyake et al. 2024; Miyake and Yozu 2025; Miyake et al. 2026).

The model predicts that during the stance phase, the left forelimb (LFb) and right hindlimb (RHb) couplet extends through activation of dorsal (e-series) muscles in the LFb and of ventral (f-series) muscles in the RHb (Gatesy 1997; Iijima et al. 2024) (Fig. 6). In the LFb, muscles such as latissimus dorsi (LD, e1), triceps brevis intermedius (TBI, e2), triceps longus lateralis (TLL, e3), and teres major (TM, e1) are active, whereas in the RHb, adductor femoris 1 (ADD1, f1), caudofemoralis longus (CFL, f1), puboischiotibialis (PIT, f3), flexor tibialis internus 2 (FTI1, f3), and flexor tibialis externus (FTE, f3) are engaged. During the swing phase, this coordination reverses, with f-series muscles active in the LFb and e-series muscles in the RHb, propelling the limbs forward (Fig. 6). In the stance phase, sequential muscle activity progresses from FA to BC in the forelimb whereas the activity progresses from BC to DE in the hindlimb (Fig. 6). In the swing phase, on the other side, sequential muscle activity progresses from BC to EF in the forelimb whereas the activity progresses from DE to AB in the hindlimb (Fig. 6). Therefore, combined force outputs become activated during both the swing and stance phases in the forelimbs and hindlimbs as the activity switches sequentially (Figs. 1A and 6). Specially, at the onset of the stance phase, the combined force output *Fe3* + *Fe2* at activity switch FA extends the forelimb, whereas the combined force output *Ff1* + *Ff3* at activity switch BC extend the hindlimb (Fig. 6, large arrows).

These coordinated activation patterns indicate that diagonal limb coupling in quadrupedal walking emerges from a systematic dorsoventral division of function across forelimbs and hindlimbs, consistent with the predictions of the two-joint link model (Miyake and Okabe 2022**;**  Miyake et al. 2024). During the stance phase, e-series muscles extend the forelimb while f-series muscles extend the contralateral hindlimb, generating asymmetric yet complementary force coordination within the LFb-RHb and RFb-LHb couplets. The repeated convergence of ipsilateral limbs at swing-stance transitions follows from dorsal e-series activity, which brings the limbs into proximity prior to phase exchange (Fig. 6, triangle marks).

We further propose that specific muscles undergo eccentric contraction (EC) within this coordination pattern. During the stance phase, the pectoralis (PEC, f1) of the forelimb and iliotibialis (ILTIB1, ILTIB2, e3), femorotibialis (FMTI, f3), and flexor tibialis externus (FTE, f3) of the hindlimb are expected to act eccentrically as the limbs extend (Fig. 6). During the swing phase, the teres major (TM, e1) and triceps longus lateralis (TLL, e3) of the forelimb, and the puboischiotibialis (PIT, f3) of the hindlimb, are similarly predicted to undergo eccentric contraction. Such coordination is consistent with stretch-shortening cycles (SSCs) observed in vertebrate locomotion, in which muscles alternate between eccentric and concentric contraction to enhance force production and efficiency (Miyake et al. 2024**;**  Miyake and Yozu 2025). SSCs have been widely documented in activities such as pedaling, sprinting, football, and cycling (Enoka 1996; Baba et al. 2000; Fukutani et al. 2020). At the molecular level, the elastic protein titin contributes to force enhancement during eccentric phases (Herzog 2014; Nishikawa 2016) and is proposed to play a key role in energy storage and force regulation during vertebrate limb locomotion (Herzog et al. 2016; Linke 2023). Together, these findings indicate that diagonal-couplet lateral sequence gait is not merely a temporal coordination pattern, but a mechanically structured system arising from the intrinsic dorsoventral organization (e-series and f-series muscles) of limb musculature, as formalized in the two-joint link model.

### Lumbar lateral motor column (LMCl) organzation and spinal basis of limb coordination

#### Topographic organization of the cat lumbar lateral motor column (LMCl)

**Fig. 7:**
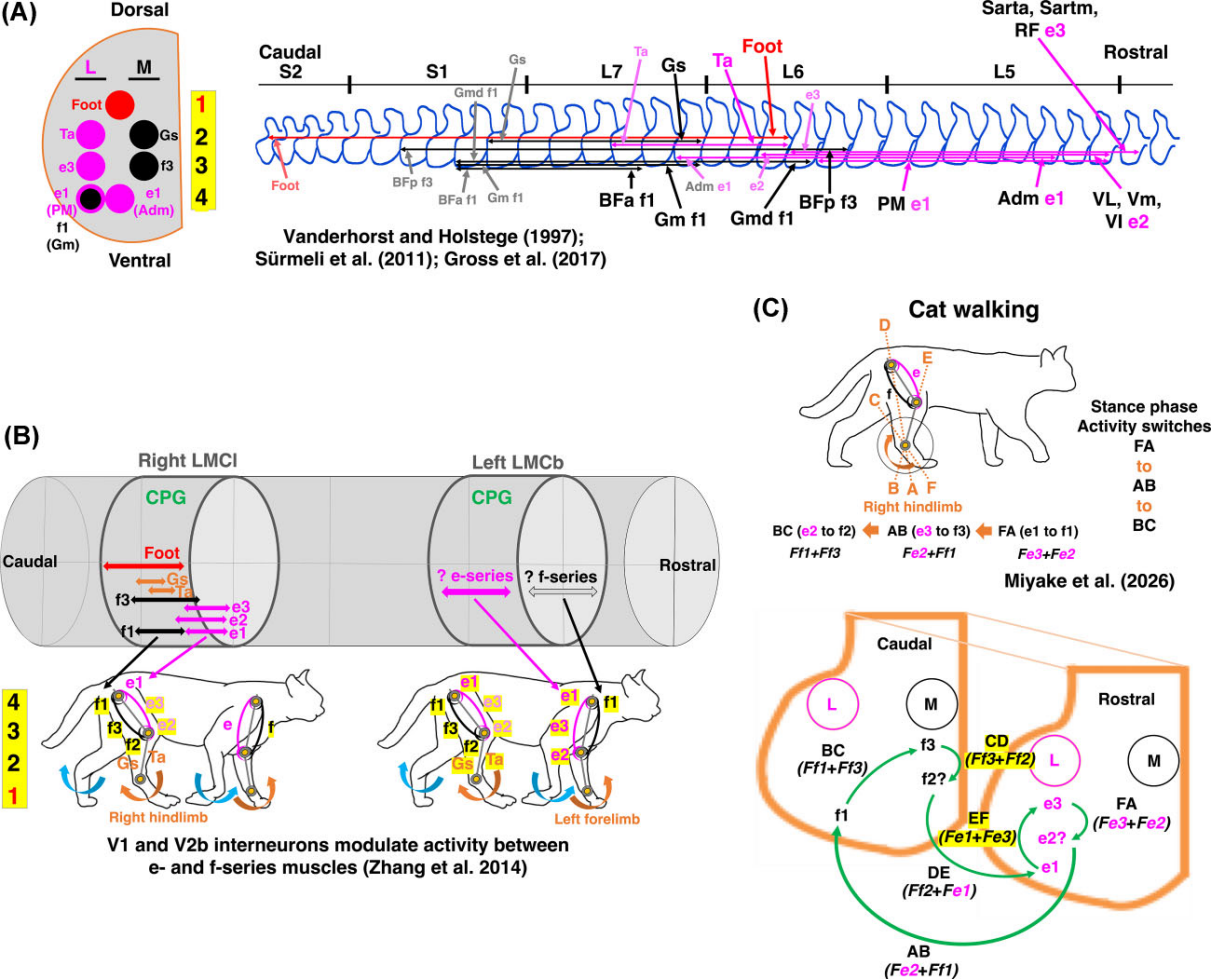
The diagonal-couplet lateral sequence gait and neural networks in central nervous system supporting limb locomotion. (A) The re-mapped locations of motor neurons in the LMCl that innervate the femoral muscles of the right hindlimb in domestic cats. The mapping is based on original data from Vanderhorst and Holstege (1997), Sürmeli et al. (2011) and Gross et al. (2017). (B) Locations of motor neurons in the lumbar lateral motor column (LMCl) of the spinal cord and their relationships with femoral muscles in the domestic cat. They are based on re-mapped analyses of Vanderhorst and Holstege (1997), Sürmeli et al. (2011) and Gross et al. (2017). (C) Cat walking and a potential motor-neuron activity pattern illustrating activity switches and the resulting combined force outputs in the LMCl. The biarticular and monoarticular muscles of the right hindlimb in cat undergo activity switches from FA to AB to BC, with the predicted combined force shifting from *Fe3 + Fe2* to *Fe2 + Ff1* to *Ff1 + Ff3* at the transition from the swing phase to stance phase during walking (Miyake et al. 2026). The cat hindlimb muscles are as follows: *M. anterior biceps femoris* (BFa, f1), *M. gluteus maximus* (Gm, f1), *M. gluteus medius* (Gmd, f1); *M. posterior biceps femoris* (BFp, f3), *M. adductor magnus* (Adm, e1), *M. psoas major* (PM, e1), *M. vastus intermedius* (VI, e2), *M. vastus medialis* (Vm, e2), *M. vastus lateralis* (VL, e2), *M. sartorius anterior* (Sarta, e3), *M. sartorius medialis* (Sartm, e3), *M. rectus femoris* (RF, e3), *M. gastrocnemius* (Gs), *M. tibialis cranialis* (Ta) and foot muscles (Foot). CPG: central pattern generator; L: lateral motor pool of LMC; L4–L7: vertebral segments of lumbar 4 to 7; LMCb: brachial lateral motor column; LMCl: lumbar lateral motor column; M: medial motor pool of LMC; S1 and S2: vertebral segments of sacral 1 and 2. Supplementary data 4.

The locomotion of diagonally coupled forelimbs and hindlimbs in tetrapods relies on coordinated activity within spinal neural circuits, particularly the lateral motor columns (LMCs), whose motor neurons innervate limb muscles and shape rhythmic locomotor output (Fetcho 1987; Rossignol et al. 2006; Murakami and Tanaka 2011; Grillner and Manira 2020). In the hindlimb, the lumbar lateral motor column (LMCl) exhibits a rostrocaudal and mediolateral organization that is thought to underlie orderly muscle recruitment during gait (Fetcho 1987; Bonanomi and Pfaff 2010; Sürmeli et al. 2011; Stifani 2014; Jung and Dasen 2015; Lu et al. 2015; D’Elia and Dasen 2018; Ferreira-Pinto et al. 2018; Osseward II and Pfaff 2019; Grillner and Manira 2020). Figure 7A illustrates the distribution of motor neurons innervating femoral and distal hindlimb muscles in the domestic cat, based on a reanalysis of anatomical and neurological datasets (Vanderhorst and Holstege 1997; Sürmeli et al. 2011; Gross et al. 2017). These studies provide the most comprehensive characterization of hindlimb motor neuron topography in the LMCl currently available.

Building on these datasets, we reexamined the spatial organization of motor neuron pools innervating biarticular and monoarticular muscles of the right hindlimb, with the aim of interpreting electromyography (EMG)-based activity switches and force outputs within the framework of the two-joint link model. We reconstructed the LMCl and remapped the corresponding motor neuron pools onto a unified schematic (Fig. 7A; Supplementary data 4). The reanalyzed muscles include *M. psoas major* (PM, e1), *M. adductor magnus* (Adm, e1), *M. gluteus maximus* (Gm, f1), *M. gluteus medius* (Gmd, f1), *M. anterior biceps femoris* (BFa, f1), *M. vastus lateralis* (VL, e2), *M. vastus medialis* (Vm, e2), *M. vastus intermedius* (VI, e2), *M. sartorius anterior* (Sarta, e3), *M. sartorius medialis* (Sartm, e3), *M. rectus femoris* (RF, e3), *M. posterior biceps femoris* (BFp, f3), *M. tibialis cranialis* (Ta), *M. gastrocnemius* (Gs), and intrinsic foot muscles (Fig. 7A). Muscle nomenclature follows primarily *the Nomina Anatomica Veterinaria*, with minor adjustments consistent with Vanderhorst and Holstege (1997) and Sürmeli et al. (2011).

#### Conserved medial-lateral motor pool segregation across cat hindlimbs

Across the lumbar lateral motor column (LMCl), motor neurons innervating e-series muscles are positioned more rostrally, whereas those innervating f-series muscles tend to occupy more caudal regions. Additionally, e-series and f-series motor pools exhibit mediolateral segregation (Fig. 7A, Supplementary data 4). Within individual LMCl sections, e-series muscles, except for *M. adductor magnus* (Adm, e1), are located predominantly in medial motor pools (M), while f-series muscles, except for *M. gluteus maximus* (Gm, f1), are located mainly in lateral motor pools (L). Notably, *M. adductor magnus* has been observed to function as an f1 muscle during human sprinting, indicating functional reassignment under task-specific conditions (Miyake and Yozu 2025). Motor neurons are further organized along the dorsoventral axis, reflecting the anatomy of hindlimb muscles: motor neurons for e3- and f3-series muscles are located dorsally, while those for e1- and f1-series muscles occupy more ventral positions (Fig. 7A, B, 1 through 4). Motor neurons innervating distal muscles, including *M. tibialis cranialis* and *M. gastrocnemius*, are concentrated in the most caudal and dorsal regions of the LMCl (Fig. 7A, B).

Across vertebrates, motor pools within the LMC exhibit a conserved medial-lateral segregation that broadly corresponds to flexor-extensor functional domains. Comparative and developmental studies across vertebrates support the principle that lateral motor pools innervate extensor muscles on the dorsal domain of the limb, whereas medial motor pools innervate flexor muscles on the ventral domain (Fetcho 1987; Francius et al. 2013; Jung and Dasen 2015; Grillner and Manira 2020) (Fig. 7B). However, because relatively few studies have examined the brachial lateral motor column (LMCb), two conflicting interpretations have been proposed. One suggests that lateral motor pools (L) innervate ventral flexor muscles and medial pools (M) dorsal extensor muscles (Pearson and Gordon 2013), whereas the other proposes the opposite arrangement (McKenna et al. 2000; Tosolini and Morris 2012; Hollis et al. 2016; Qi et al. 2022). Recent experimental evidence from macaques resolves this discrepancy, demonstrating that lateral motor pools (L) innervate dorsal extensor muscles and medial motor pools (M) innervate ventral flexor muscles in the forelimb as well (Taitano et al. 2024). These findings, combined with our remapping of the LMCl, support a unified organizational framework for forelimb and hindlimb motor control (Miyake and Okabe 2022; Miyake et al. 2024).

#### Neuromechanical interpretation of activity switches in cat walking

Based on the two-joint link model, Miyake et al. (2026) examined and reviewed previously published electromyographic (EMG) data of the right hindlimb in cat walking and compared the results with those of dogs, horses, and humans. In cat walking gait, specifically, at the transition from stance to swing, the right hindlimb becomes flexed through activation of *M. psoas major* (PM, e1) and *M. sartorius* (Sart, e3), accompanied by eccentric contraction of *M. posterior biceps femoris* (BFp, f3). This transition corresponds to an activity switch in the sector EF, with the predicted combined force *Fe1 + Fe3* (Fig. 7C). Conversely, during the transition from swing to stance, *M. gluteus maximus* (Gm, f1), *M. gluteus medius* (Gmd, f1), *M. anterior biceps femoris* (BFa, f1), and *M. posterior biceps femoris* (BFp, f3) are coactivated with eccentrically activated *M. vastus muscles* (VL, Vm, VI; e2), extending the right hindlimb. This phase corresponds to activity switches from FA to AB to BC, with the predicted combined force shifting from *Fe3 + Fe2* to *Fe2 + Ff1* to *Ff1 + Ff3* (Miyake et al. 2026) (Fig. 7C). Together, these results indicate that the spatial organization of LMCl motor pools constrains activity switches and combined force outputs, thereby shaping limb mechanics during walking and supporting robust, evolutionarily conserved patterns of tetrapod locomotion. Importantly, the combined force outputs at the onset of stance (*Ff1* + *Ff3*) and swing (*Fe1* + *Fe3*) are similar in American alligators and cats (Miyake et al. 2026) (Fig. 6). This correspondence suggests a shared neuromechanical strategy, whereby forelimb extension is predominantly mediated by e-series muscles, whereas hindlimb extension is driven by f-series muscles.

#### Spinal circuit integration: central pattern generators and interneurons

The proposed pattern of activity switches likely emerges from interactions among motor neurons, spinal interneurons, and central pattern generator (CPG) circuits. Commissural interneurons, together with V1 and V2b interneurons, regulate ipsilateral and bilateral coordination, while afferent sensory inputs including force-sensitive Golgi tendon organ feedback modulate task-specific motor output during walking (Grillner and Zangger 1979; Kiehn 2006; Frigon 2017; Miyake and Okabe 2022) (Fig. 7B). CPGs within the spinal cord generate the fundamental locomotor rhythm autonomously, independent of supraspinal commands (Grillner 1975; Grillner 2006; Kiehn 2006; Kiehn 2011), and propriospinal neurons linking cervical and lumbar segments facilitate synchronized interlimb coordination in quadrupedal mammals (Juvin et al. 2005). Elucidating how these circuit elements interface with the spatial organization of LMCl motor pools is therefore essential for understanding the neurophysiological basis of muscle coordination and force control during tetrapod locomotion.

Within this framework, the spatial topography of LMCl motor pools provides a plausible neural substrate for the coordinated activity switches and muscle coactivations observed during cat walking gait (Fig. 7C). We propose that sequential activation across distinct LMCl motor pools generates the combined force outputs predicted by the two-joint link model (Miyake and Okabe 2022). At each activity transition, such as from e3 to f3, specific pairs of biarticular and monoarticular muscles are recruited from different motor pools, enabling systematic redirection of endpoint forces across defined sectors of the ankle joint (Fig. 7C). These transitions correspond to EMG-derived stance–swing patterns and support a neuromechanical interpretation in which motor neuron topography constrains and facilitates orderly modulation of limb forces (Miyake et al. 2026).

Interneurons within the LMC, including V1 and V2b interneurons, contribute to switching activity between antagonistic muscle groups, such as e-series and f-series muscles, ensuring appropriate force transmission during gait (Zhang et al. 2014; Grillner and Manira 2020). Commissural interneurons (CINs), particularly V3 interneurons, further mediate coordination of ipsilateral and contralateral limb movements, a defining feature of complex gaits such as the diagonal-couplet lateral sequence (Danner et al. 2019; Shevtsova et al. 2022). The LMC thus functions as an integrative node that orchestrates patterned muscle recruitment in accordance with CPG-driven rhythms, providing the spinal foundation for adaptive limb locomotion across vertebrates.

## Discussion

The two-joint link model provides a mechanistic framework for understanding coordinated interactions between biarticular and monoarticular muscles across human and non-human tetrapod locomotion. Developed through experimental, mechanical, and theoretical investigations (Fujikawa et al. 1996; Fujikawa et al. 1997; Oshima et al. 1999; Fujikawa et al. 2001; Oshima 2008), the model formalizes three antagonistic muscle pairs and predicts sequential activity switches that systematically reorganize intersegmental force transmission into structured endpoint force outputs (Miyake and Okabe 2022; Miyake et al. 2024; Miyake and Yozu 2025). These predicted transitions are consistent with electromyographic (EMG) data and are observed across locomotor tasks including walking, pedaling, and sprinting in humans, as well as quadrupedal walking in species such as domestic cats and the American alligator (Gatesy 1997; Iijima et al. 2024; Miyake et al. 2024; Miyake and Yozu 2025; Miyake et al. 2026).

Comparative observations from extant taxa further contextualize this framework. The coelacanth *Latimeria* and the American alligator *Alligator mississippiensis* exhibit coordinated diagonal-couplet fin and limb phasing, respectively, that is compatible with the alternating force redirection predicted by the model. Fossil trackways from the Devonian and Permian suggest patterns that may reflect alternating contralateral forelimb-hindlimb coordination in early tetrapods (Niedźwiedzki et al. 2010; Nyakatura et al. 2019). Nevertheless, such ichnological evidence reflects inferred kinematic states rather than providing direct evidence of muscle activity. Accordingly, while the persistence of diagonal-couplet patterns in extant archosaurs is consistent with evolutionary continuity, the underlying neuromuscular mechanisms in ancestral forms remain inferential rather than directly observable.

Human locomotion provides an additional functional perspective. Diagonal-couplet coordination is observed across crawling, walking, running, and sprinting, (Struble and Gibb 2022; Miyake and Yozu 2025). During sprint acceleration, the hindlimb exhibits phase-dependent muscle activity, including early-phase activation prior to ground contact, consistent with sequential switching among antagonistic muscle pairs, while coordinated transitions between f-series and e-series activity in the contralateral limb are consistent with the predicted redirection of combined force outputs (Miyake and Yozu 2025). Forelimb motion contributes to horizontal force production and interacts with hindlimb reflex modulation through spinal circuitry (Frigon et al. 2004; Nezu 2023). Collectively, these observations support the interpretation that interlimb coordination arises from coordinated force redistribution mediated by structured muscle switching within spinal circuits, rather than from isolated joint-level control.

From a biomechanical standpoint, the two-joint link model highlights the functional importance of dorsoventral muscle domain organization expressed through the e-series and f-series. These series represent coordinated functional groupings defined by their roles in intersegmental force transmission, rather than fixed flexor-extensor identities. In forelimbs, stance-phase extension is predominantly generated by e-series muscles, whereas in hindlimbs, extension is largely produced by f-series muscles (Miyake and Okabe 2022; Miyake et al. 2026). This complementary dorsal-ventral distribution creates alternating rotations of resultant force outputs across the shoulder-elbow and hip-knee linkages. Consequently, diagonal-couplet coordination is interpreted not merely as a temporal phase relationship but as a mechanically structured oscillation of endpoint force orientation produced by sequential switching between antagonistic muscle series. Within this framework, apparent extension or flexion emerges as a context-dependent outcome of coordinated force transmission across linked joints, rather than as an intrinsic property of individual muscles.

Thus, gait can be understood as an emergent property of linkage-based force organization. At the same time, the present framework has defined scope boundaries. The two-joint link model primarily addresses the organization and redirection of endpoint forces generated by coordinated muscle activation across paired appendages. It does not explicitly account for large-scale postural transitions in tetrapod evolution, such as the shift from sprawling limb postures to more upright parasagittal configurations, which involve additional skeletal and kinematic reorganizations of the appendicular system (Gatesy 1997; Nyakatura et al. 2019). Detailed analyses of limb mechanics in extant taxa likewise highlight the contribution of complex musculoskeletal interactions that extend beyond simplified planar abstractions (Iijima et al. 2024). Accordingly, the model should be interpreted as a first-order biomechanical framework for organizing limb-level force coordination and intersegmental coupling, rather than a complete reconstruction of three-dimensional musculoskeletal function or evolutionary transformation.

## Conclusion

Comparative application across extant tetrapods and sarcopterygian lineages suggests that the fin-to-limb transition was not a simple structural replacement but a progressive reorganization of pre-existing biomechanical principles governing force transmission and redirection. Controlled fin rotation in early tetrapod morphs likely functioned as a precursor to limb-like patterns of force transmission and redirection (Niedźwiedzki et al. 2010; Miyake et al. 2016), consistent with shifts in dorsoventral limb patterning (Zdra et al. 2025). These changes suggest that fin-based force redirection established a functional foundation for subsequent limb-level coordination structured by e-series and f-series muscle activity. Although fossil morphology and trackway evidence remain inferential, integration of electromyographic (EMG) data with model-based force predictions supports the hypothesis that diagonal-couplet neuromuscular organization may predate fully developed limbs (Gatesy 1997; Iijima et al. 2024; Miyake and Yozu 2025). Accordingly, coordinated dorsoventral muscle switching may represent a conserved vertebrate strategy for organizing endpoint forces across paired appendages rather than a purely terrestrial innovation.

At its core, the two-joint link model identifies musculoskeletal redundancy as a fundamental vertebrate design principle: multiple muscle combinations can generate equivalent functional outputs, enhancing stability, adaptability, and evolutionary flexibility (Oshima et al. 2000; Obinata et al. 2003; Almanzor et al. 2024). This redundancy arises from the four-bar linkage organization of biarticular muscle systems, in which geometric coupling constrains force transmission while permitting multiple activation patterns. Within this framework, functional outputs are preserved despite variability in individual muscle contributions, reflecting a system-level organization of force rather than one-to-one muscle-function mapping. Comparable forms of functional redundancy occur in other biomechanical systems, including the four-bar linkage mechanisms underlying fish cranial kinesis, where diverse morphological configurations yield similar mechanical outputs and permit evolutionary diversification while preserving effective force transmission (Alfaro et al. 2004; Arbour et al. 2024).

Limb evolution reflects, therefore, not only anatomical transformation but also the conservation and redeployment of a robust mechanical logic grounded in coordinated force transmission across linked segments across vertebrate lineages. Diagonal-couplet coordination can be interpreted as a persistent expression of this linkage-based force architecture, maintained under diverse postural and environmental conditions. Viewed in this broader comparative context, the two-joint link model captures a general vertebrate design principle linking redundancy, robustness, and evolvability, and provides a unifying framework for understanding muscle coordination, force generation, and the evolutionary logic of locomotor mechanics across vertebrate lineages. Future comparative studies integrating musculoskeletal modeling, electromyography, and fossil-informed reconstruction will be important for testing how linkage-based force organization is maintained or modified across different mechanical and evolutionary contexts.

## Supplementary Material

obag020_Supplemental_Files

## Data Availability

All data pertaining to this manuscript are included in the body of the text and in the source publications cited, as Supporting Information files.
