# Supplementary material for: From Fin to Limb: Orientational Shift and Evolution of Diagonal-Couplet Gait in Tetrapods
Source: Integr Org Biol. 2026 May 6;8(1):obag020. doi: 10.1093/iob/obag020 (PMC13199859; doi:10.1093/iob/obag020)
Supplement: obag020_Supplemental_Files [file obag020_supplemental_files.zip › Supplementary data 1.pdf]

Supplementary data 1

From fins to limbs: orientational shift and evolution of diagonal-couplet gait in tetrapods.

Tsutomu Miyake, Kanto Nishikawa, Masamitsu Iwata, Hiroko Kamiyama, Kohtaro Ozaki, Hiroshi Koie, Arito Yozu, Tetsuya Hirasawa and Naoto Kobayashi.

Movements of pectoral and pelvic fins in *Latimeria chalumnae* and *L. menadoensis*:

Miyake T, Kumamoto M, Iwata M, Sato R, Okabe M, Koie H, Kumai N, Fujii K, Matsuzaki K, Nakamura C, Yamaguchi S, Yoshida K, Yoshimura K, Komada A, Uyeno T, Abe Y. 2016. The pectoral fin muscles of the coelacanth *Latimeria chalumnae*; functional and evolutionary implications for the fin-to-limb transition and subsequent evolution of tetrapods. Anat Rec 299:1203-1223.

https://doi.org/10.1002/ar.23392.

Page 1208: Fig 3 – Anatomy of pectoral fin

Page 1215: Fig. 9 - Activity of pectoral fin muscles.

Page 1218: Fig.12 - A summary of the pectoral fin muscles

Iwata M. 2017. Possible existence of unified motion control principles in the animal kingdom: biological and engineering analyses of coelacanth swimming. PhD. thesis. College of Enginering, Kanazawa Institute of Technology, Ohgigaoka Nonoichi, Ishikawa, Japan. In Japanese.

http://kitir.kanazawa-it.ac.jp/infolib/cont/01/G0000002repository/000/000/000000143.pdf.

Page 30 ~ 33: Structure and geometric dimensioins of the pectral fin

Page 30 ~ 40 : Kinematic analysis of the actual coelacanth

Page 34: Fig. 18 - Relative positioning of the fins

Page 36: Fig. 21 - Temporal variation of the pitch angle beta for P1R, P1L, D2 and A1.
