# Supplementary material for: From Fin to Limb: Orientational Shift and Evolution of Diagonal-Couplet Gait in Tetrapods
Source: Integr Org Biol. 2026 May 6;8(1):obag020. doi: 10.1093/iob/obag020 (PMC13199859; doi:10.1093/iob/obag020)
Supplement: obag020_Supplemental_Files [file obag020_supplemental_files.zip › Supplementary data 2.pdf]

Supplementary data 2

From fins to limbs: orientational shift and evolution of diagonal-couplet gait in tetrapods.

Tsutomu Miyake, Kanto Nishikawa, Masamitsu Iwata, Hiroko Kamiyama, Kohtaro Ozaki, Hiroshi Koie, Arito Yozu, Tetsuya Hirasawa and Naoto Kobayashi.

The foot prints or trackways of tetrapod fossils:

Niedźwiedzki G, Szrek P, Narkiewicz K, Narkiewicz M, Ahlberg PerE. 2010. Tetrapod trackways from the early Middle Devonian period of Poland. Nature 463:43-48.

https//doi.org/10.1038/s41586-018-0851-2.

Page 45: Fig. 2 - Trackways.

Nyakatura, JA, Melo K, Horvat T, Karakasiliotis K, Allen VR, Andikfar A. et al. 2019. Reverse-engineering locomotion of a stem amniote. Nature 565:351-355.

Extended Data Fig. 2: Kinematic simulation of Orobates.
