# Supplementary material for: From Fin to Limb: Orientational Shift and Evolution of Diagonal-Couplet Gait in Tetrapods
Source: Integr Org Biol. 2026 May 6;8(1):obag020. doi: 10.1093/iob/obag020 (PMC13199859; doi:10.1093/iob/obag020)
Supplement: obag020_Supplemental_Files [file obag020_supplemental_files.zip › Supplementary data 3.pdf]

Supplementary data 3

From fins to limbs: orientational shift and evolution of diagonal-couplet gait in tetrapods.

Tsutomu Miyake, Kanto Nishikawa, Masamitsu Iwata, Hiroko Kamiyama, Kohtaro Ozaki, Hiroshi Koie, Arito Yozu, Tetsuya Hirasawa and Naoto Kobayashi.

The electromyographic (EMG) data on the hindlimb in American alligators:

Gatesy SM. 1997. An electromyographic analysis of hindlimb function in Alligator during terrestrical locomotion. J Morph 234:197-212. https://doi.org/10.1002/(SICI)1097- 4687(199711)234:2<197::AID-JMOR6>3.0.CO;2-9.

Page 201: Fig. 2 - Anatomy of muscles.

Page 202: Fig. 3 - Anatomy of muscles

Page 203: Fig. 4 - Electromyography of hindlimb muscles

Page 204: Fig. 5 - Summary of electromyography

The electromyographic (EMG) data on the forelimb in American alligators:

Iijima M, Mayer CJ, Munteanu VD, Blob RW. 2024. Forelimb muscle activation patterns in American Alligators: insights into the evolution of limb posture and powered flight in archosaurs. J Anat 244:943-958. https://doi.org/10.1111/joa.14011.

Page 946: FIGURE 1 - Anatomy of the forelimb muscles

Page 949: FIGURE 2 - Electromyography of forelimb muscles

Page 950: FIGURE 3 - Electromyography of forelimb muscles with strike cycle
