# Supplementary material for: From Fin to Limb: Orientational Shift and Evolution of Diagonal-Couplet Gait in Tetrapods
Source: Integr Org Biol. 2026 May 6;8(1):obag020. doi: 10.1093/iob/obag020 (PMC13199859; doi:10.1093/iob/obag020)
Supplement: obag020_Supplemental_Files [file obag020_supplemental_files.zip › Supplementary data 4.pdf]

Supplementary data 4

From fin to limb: orientational shift and evolution of diagonal-couplet gait in tetrapods.

Tsutomu Miyake, Kanto Nishikawa, Masamitsu Iwata, Hiroko Kamiyama, Kohtaro Ozaki, Hiroshi Koie, Arito Yozu, Tetsuya Hirasawa and Naoto Kobayashi.

Cat lateral motor column (LMC):

The distribution of motor neurons in domestic cat lateral motor column:

VanderHorst VG, Holstege G. 1997. Organization of lumbosacral motoneuronal cell groups innervating hindlimb, pelvic floor, and axial muscles in the cat. J Comp Neur 382:46-76.

https://doi.org/10.1002/(SICI)1096-9861(19970526)382:1<46::AID-CNE4>3.0.CO;2-K

Figs 1 ~ 26. The map of the motor neurons in the lateral motor column.

Sürmeli G, Akay T, Ippolito GC, Tucker PW, Jessell TM. 2011. Patterns of spinal sensory-motor connectivity prescribed by a dorsoventral positional template. Cell 147:653-665.

https://www.cell.com/cell/fulltext/S0092-8674(11)01210-4?_returnURL=https%3A%2F%2Flinkinghub.elsevier.com%2Fretrieve%2Fpii%2FS0092867411012104%3Fshowall%3Dtrue

Page 654: Figure 1 - The distribution of motor neurons in LMCl and the hindlimb muscles.

Gross C, Ellison B, Buchman AB, Terasawa Ei, VanderHorst VG. 2017. A novel approach for assigning levels to monkey and human lumbosacral spinal cord based on ventral horn morphology. PLoS One 12:1-13.

https://journals.plos.org/plosone/article?id=10.1371/journal.pone.0177243

Page 8: Figure 3 - Schematic drawings of lumbosacral sections in cat, monkey and human aligned via internal landmarks.
